# The Axon as a Self-Modifying Computational System: Autonomous Inference, Adaptive Propagation, and AI-Enabled Mechanistic Insight

**DOI:** 10.3390/ijms27041826

**Published:** 2026-02-14

**Authors:** Matei Șerban, Corneliu Toader, Răzvan-Adrian Covache-Busuioc

**Affiliations:** 1 Department of Neurosurgery, “Carol Davila” University of Medicine and Pharmacy, 050474 Bucharest, Romania; 2 Department of Vascular Neurosurgery, National Institute of Neurology and Neurovascular Diseases, 077160 Bucharest, Romania; 3 Puls Med Association, 051885 Bucharest, Romania

**Keywords:** axonal computation, adaptive propagation, metabolic microdomains, electromechanical signaling, ultrastructural dynamics, physics-informed modeling, AI-driven connectomics, closed-loop neuromodulation, myelin plasticity, subcellular computation

## Abstract

Research has demonstrated that axonal signaling processes are influenced by both static structural factors and dynamic metabolic and electro-dynamic processes. Imaging, computational modeling and research in molecular neuroscience have demonstrated that multiple processes contribute to axonal signal processing, including periodic rearrangement of cytoskeletal structures and membrane structures, and redistribution of ion channel clusters and organelles (such as mitochondria), which occur rapidly and transiently to modify excitability. The dynamics of energy production and distribution also vary between regions of the axon and at different time points during signal generation and transmission. Additionally, myelin-associated glia may temporarily modulate their metabolic and structural contributions to axonal conduction. Advanced AI-based techniques for mapping and simulating ultrastructure and the use of closed-loop perturbation experiments demonstrate that axons can generate multiple distinct electromechanical states, and therefore potentially influence both the timing of signals generated by the axon, the routing of signals to branches of the axon, and the robustness of signal propagation. While the existence of these adaptive microstates appears well established, there are many aspects of their influence on circuit level function that are poorly understood. In summary, these data support the concept that axonal conduction represents a continuum of reversible and state-dependent configurations generated by integrated interactions among molecular, structural and energetic processes. Therefore, this review will attempt to synthesize the available literature into a unified conceptual framework and identify areas of uncertainty that may direct future research into the adaptive processes underlying axonal computation.

## 1. Introduction

Traditionally, the axon was thought of merely as a passive pathway for the propagation of action potentials. However, recent evidence indicates that the axon is far more complex than this simple model suggests and that the traditional view of the axon can no longer account for all of the functional diversity found in the various types of axons. Axons, like dendrites, can regulate excitability through local mechanisms in addition to molecular and biophysical determinants of conduction. Depending on the environment, axons can regulate conduction fidelity and synaptic output. One example of an axon’s ability to function independently is the process of proteostasis, in which axons can translate RNA into proteins for the regulation of ion channels, cytoskeletal dynamics and metabolism. Local translation of proteins within the axon can be regulated through depolarization, increased intracellular calcium, and trophic factors, indicating that rapid localized responses can occur within the axon to external stimuli [1,2]. In addition to rapid localized responses, it is possible that the local translation of proteins within the axon serves as a form of division of labor with the soma, serving as a source of basal proteins and the axon serving as a source of “on-demand” modifications in response to local “state variables” such as, but not limited to, activity history, calcium levels, trophic factors, and energy reserves [3,4,5]. Consistent with this concept, it has been demonstrated that proteins that serve as regulators of sodium channels (Nav1.6) and potassium channels (KCQN), as well as proteins that maintain the stability of microtubules, are present in areas of the axon that are capable of generating action potentials [6]. Additionally, ribosomal clusters have been shown to localize close to mitochondria, suggesting that local ATP availability may impact translation capability [7].

In addition to the distribution of proteins along the length of the axon, compartmentalization of the axon can also contribute to the generation of axonal signaling. The degree of compartmentalization can determine both whether an action potential will propagate and how the waveform and timing of action potentials will develop during propagation [8]. The implications for understanding how downstream synaptic transmission fidelity is influenced by different patterns of synaptic drive are significant [9]. Similarly, the axon initial segment (AIS) determines the threshold at which an action potential will initiate and the shape of the first few milliseconds of the action potential’s waveform, which in turn affect downstream conduction fidelity. Furthermore, the presynaptic terminal transforms the dynamic processes of propagation into the release of a neurotransmitter through terminal-specific channel composition, calcium handling and release machinery, thus linking the dynamic processes of propagation to synaptic efficacy and plasticity [10].

Additionally, the rate of propagation and the shape of the action potential waveform can depend on the preceding activity, the local ionic concentrations, sodium channel inactivation, and the conductance–capacitance ratio of the area of interest, leading to measurable differences in latency, spike width and the morphology of the action potential afterpotential [11]. Alterations in the biophysics of action potential conduction can alter the precision of spike timing and, consequently, the amount of vesicle released, providing a direct link between the microdomains of the axon and circuit-level computations [12,13,14]. Activity-dependent post-translational modification of axonal microtubules (i.e., acetylation and polyglutamylation) can regulate interactions with motor proteins and redistribute mitochondria and endosomes, and, therefore, can influence the trafficking of cargo to regions of high demand [15]. Activity-dependent gradients of phosphorylated neurofilaments can create gradients of neurofilament spacing, axonal diameter, and axial resistance, and therefore modulate conduction properties for an extended period of time [16].

Recent super-resolution imaging studies suggest that many of the key channels, including Nav1.6 and Kv-family members, exist as patterned nanodomains that regulate action potential kinetics and recovery, and repeated activity can lead to changes in the nanodomains and subsequent alterations in excitability and high-frequency firing [17]. Conduction can also be altered by heterogeneity in HCN-channel density, nodal spacing, local Ca^2+^ dynamics, extracellular K^+^ buffering, ephaptic interactions and stochastic channel gating, highlighting the dependence of local microenvironmental conditions [18,19].

The relationship between metabolic regulation and these mechanisms provides yet another important connection. The redistribution of mitochondria is dependent on Ca^2+^ transients and ATP/ADP ratios, and redistributions can modulate redox states, glycolytic fluxes and recovery of Na^+^/K^+^-ATPase, thereby altering refractory periods and the reliability of repetitive firing [20]. Myelination also provides a slow regulatory axis; activity-dependent changes in internode lengths, sheath thickness and paranodal organization can modulate the timing of conduction and may provide a basis for coordinated activity across distributed networks [21].

Concurrently, AI-based methodologies are rapidly improving their capacity to map and simulate the relationships between the structure and function of axons, including deep learning-based segmentation of ultrastructure; multi-modal feature extraction; generative reconstruction of long-range morphological profiles; physics-informed simulation of conduction in a variety of constrained environments; and adaptive stimulation paradigms that can query excitability landscapes in real-time [22,23,24]. Collectively, the findings reported herein indicate that the axon should be considered as a self-contained signaling entity whose characteristics emerge from the interplay among molecular composition, nanoscale organization, energetic state and local electrodynamics, while acknowledging that the degree to which these adaptations are independent of somatic and network-level regulation remain unknown [25].

This review has summarized biochemical, structural, metabolic and computational advancements relevant to axonal autonomy, highlighted gaps in current knowledge, and described how AI-based mapping and closed-loop interrogation may enable the identification of causal control variables governing propagation, robustness, and susceptibility to failure.

## 2. Molecular and Structural Bases of Axonal Autonomy

### 2.1. Local Translatomes and Spatially Resolved Protein Synthesis

Axons contain the machinery needed to synthesize proteins at specific locations and at different times, which allows them to adapt to changes in their environment and physiology. Ribosomes have been shown to be found in axons and can have different combinations of subunits (e.g., RPL38-, RPS25-, and RPS29-like enriched). This could allow axonal ribosomes to preferentially translate mRNA containing specific 5′ untranslated regions or unique codon usage [26]. Ribosomal RNA pseudouridylation via the action of PUS enzymes has also been reported to alter the efficiency of decoding in a context-specific manner [27].

Axonal mRNAs are regulated by features such as G-quadruplex motifs, N6-methyladenine (m6A), and cap-associated methylation (m6Am). Additionally, axonal mRNAs are bound by RNA-binding proteins (RBPs) (e.g., FMRP, G3BP1, hnRNPA3, and LARP4B) into transportable RNA granules that can target specific regions of the neuron where translation is desired [28,29].

Localized translation is controlled by both local signaling architecture (submicron scale) and long-range signals (axoneme scale): EEF2K- and mTOR-independent nanoclusters can create localized zones of either high or low translation potential, whereas AMPK is activated when the cellular energy status is decreased (i.e., lower ATP:ADP ratio), and thus regulates protein synthesis in proportion to available energy. Spatially localized calcium sources (L-type, Orai, and IP3R3) are used to recruit ribosomes to translation sites, providing a second mechanism for controlling translation potential [30,31,32].

Finally, evidence suggests that the tRNA pool present in the axon, as well as tRNA-modifying enzymes that localize to different regions of the axon (e.g., TRMT61A and TRMT10C), may result in variations in the elongation rate and optimal codon usage along the length of the axon, suggesting that there are two additional layers of regulation that control the expression of proteins along the length of the axon [33,34].

In combination, these mechanisms support localized, cue responsive proteomic states along axons [35].

### 2.2. Cytoskeletal Plasticity and Long-Term Structural Encoding

Increasingly, the axonal cytoskeleton is recognized as a patterned, adaptive structure that encodes long-term structural constraints on signaling. The cytoskeleton consists of microtubules, which can form microdomains with regional mosaics of tubulin isoforms (e.g., TUBB3/TUBB4B with TUBA1A/TUBA1B/TUBA8 microdomains). These microdomains possess distinct mechanics and motor preferences, thereby determining the trafficking of organelles and the local structural identity [36].

Spatially organized patterns of post-translational modifications (PTMs) on tubulins —acetylation, detyrosination, Δ2-tubulin, and polyglutamylation—determine the preference of kinesins and dyneins for binding and the subsequent movement of mitochondria, lysosomes, and ER tubules; local enzymes (TTLL7, HDAC6, and vasohibin/SVBP) further allow for the tuning of each microdomain [37]. As PTMs on the cytoskeleton respond to sustained activity and metabolic stress, it has been proposed that they represent slow “load-history” substrates [38]. The spectrin–actin periodic lattice (~190 nm spacing) forms a mechanical layer that can undergo region-specific turnover and composition based upon tension and activity. Additionally, Arp2/3-dependent actin patches provide anchoring points for the trafficking of vesicles and the formation of branches [39]. Phosphorylation of neurofilaments (NF-H/NF-M tails) controls filament spacing, diameter, and stiffness, and its dynamic behavior can evolve over hours to weeks depending on the presence of calcium ions and kinases [40]. Interfaces between the cytoskeleton and organelles occur through curvature-defined ER domains and ER–mitochondria contacts (MAMs), allowing for the exchange of lipids, transfer of calcium ions, and redox coupling, which can affect excitability and branching propensity [41].

Together, the axonal cytoskeleton represents a multilayered mechanochemical system capable of establishing future signaling by encoding past states [42].

### 2.3. Phase-Separated Signaling Condensates and Subcellular Compartmentalization

Phase separation provides a rapidly reversible mechanism for organizing axonal microdomains by concentrating molecules into condensed phase structures known as mesoscale condensates that assemble and disassemble based on local conditions [43]. Kinase-scaffold condensates (CaMKIIδ, ERK1/2, PKC, AKAP scaffolds, and RAF–MEK assemblies) can transiently enhance local reaction efficiency following a Ca^2+^ influx or depolarization [44].

RNA–protein granules (FUS, TDP-43, hnRNPA2/B1, G3BP1, Ataxin-2, TIA-1 and lncRNAs) control transcript availability by altering the material properties in response to activity levels, thereby complementing the control of ribosome localization with transcript-level control [45]. Lipid-regulatory condensates (PI3K-C2β, PI4KA, PLCβ3, and ORP-family proteins) can locally modulate PIP2/PIP3 and phosphatidic acid to modify channel behavior, vesicle fusion, and branch stability [46].

Redox and metabolic condensates contain clustered glycolytic and shuttle enzymes (GAPDH, PKM2, and LDH-A), peroxiredoxins, and MAM-associated factors to produce localized ATP/redox signatures that can modify the performance of ion pumps and conduction robustness [47]. Many of these condensates interface with organelles and the cytoskeleton to maintain their position and function, and many are mechanically sensitive and will link mechanical stress to biochemical organization [48,49].

While the causal relationship between the formation of these condensates and propagation outcome has not been established, associations between condensate states and propagation outcomes suggest a possible role in regulating state-dependent axonal signaling [50,51]. Table 1 shows how spatial translation control, cytoskeletal identity coding, organelle interfaces, and condensate-based signaling work together to produce localized microenvironments contributing to axonal autonomy.

## 3. Electrodynamic Computation: Ion-Channel Microcircuits and Branch-Specific Processing

To better understand the propagation of action potentials in neurons, researchers moved beyond the idea that the speed of signal transmission was solely dependent upon the physical characteristics of the neuron and into the realm of understanding how the spatial arrangement of the microcircuits within the neuron influences the speed of signal transmission. In addition to voltage-gated sodium (Na^+^), potassium (K^+^), and calcium (Ca^2+^) channels, researchers have identified nanoscale units, which contain multiple channel types and various geometric configurations, depending on the segment of the axon, therefore influencing spike timing, waveform, and likelihood of spike failure [61,62]. These channel clusters interact with lipid microdomains, cytoskeleton scaffolding structures, and the ionic environment of the periaxonal space, therefore providing a mechanistic basis for why the action potential may develop in a significantly different fashion in various segments of the same axon. For example, repeated stimulation at a high frequency can result in significant variability in the development of the spike waveform, and if an axon has limited energy stores, the development of the spike waveform may also be affected [63].

In addition, the spatial distribution of channel clusters at the nanoscale provides the structural basis for compartmentalizing excitability along the axon. For example, the spatial distribution of Nav1.6/Nav1.2 channels that are stabilized by ankyrin-G/βIV-spectrin and PIP2-enriched domains determines the time course of the spike’s rising phase and post-spike refractory period, whereas the spatial distribution of K^+^ conductance, such as Kv1.1/Kv1.2 in juxtaparanodal regions and Kv7.2/Kv7.3 in other regions, determines both the stability of the spike and the ability to fire repetitively [64,65,66,67]. At the same time, the rapid generation of localized Ca^2+^ nanoscale domains, resulting from the spatial proximity of CaV2.x and T-type channels to sites of ER-mediated release (i.e., IP3R3/ryanodine receptors) and ion exchange systems, allow for rapid modulation of coupled Na^+^/Ca^2+^ gradients that correlate microdomain physiology to the robustness of propagation [68].

**Figure 1:**
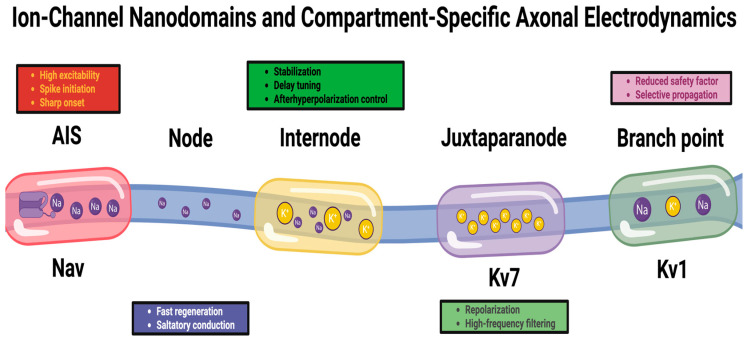
Ion-channel nanodomains and compartment-specific axonal electrodynamics. Axons contain discrete functional compartments (AIS, node, internode, juxtaparanode, and branch point) with distinct channel clustering and excitability constraints. Nav-rich domains support spike initiation/regeneration, Kv7-rich regions stabilize membrane potential and tune timing, Kv1-enriched juxtaparanodes shape repolarization and high-frequency firing, and branch points reduce safety factor, enabling conditional propagation. Together, these nanodomains shape spike waveform, timing, and reliability.

Therefore, these properties generate electrodynamic motifs in the axon that limit the flow of information in a location-dependent manner (Figure 1) [69,70].

The micro-circuitry of branching points demonstrates this conceptually, because branching points create impedance discontinuities that depend on geometry (e.g., diameter ratio of branches, tapering, and capacitance load) to define the safety margin for each pathway and the branching-specific expression of certain channels (e.g., HCN-enriched branches that support particular firing regimes vs. Kv-enriched branches that inhibit invasion of similar inputs) that can direct routing [71]. Additionally, the regulation of channel availability based on past activity (including modulation of Na^+^ channel gating) provides a mechanism for historical dependence, so that the recent activity of a branch can influence its responsiveness to subsequent inputs [72].

Finally, the microenvironment surrounding the axon adds one more layer of context dependency to the responses of branching points to stimuli: variations in the volume of the periaxonal space, how glial cells modulate K^+^, the type of contacts between microglial cells and axons, and the ephaptic interactions between densely packed axons in bundles can all contribute to changes in thresholds and selective recruitment of branches in a timing-dependent manner [12,73].

However, as the margins of safety become reduced, propagation is less predictable and more sensitive to random and microenvironmental fluctuations. Random channel-state transitions can introduce timing jitter and increase the probability of failure, effects that are amplified in smaller-caliber or unmyelinated compartments [74]. In addition, activity-dependent K^+^ accumulation and changes in Na^+^/Ca^2+^ exchanger behavior can modify local ionic gradients, while pH and redox microdynamics can slightly affect the gating and waveform development of action potentials [14,75].

Therefore, this combination defines an adaptive landscape in which the outcome of propagations can be probabilistic rather than determined, allows for flexible routing under restrictive conditions, and defines the criteria that will allow for successful conduction failure [76].

## 4. Metabolic Gating, Myelin Learning, and Propagation Plasticity

Mitochondria and myelin are now recognized as more than passive, supportive components of neural physiology. They also serve as active regulators of axonal propagation through their influence upon the availability of ATP and redox states and the geometry of electrical conduction. These slower, more enduring forms of regulation of spike timing, reliability and susceptibility to failure work together with the electrodynamic mechanisms described in Section 3 [77,78].

### 4.1. Mitochondrial Positioning, Energy Microdomains, and Activity-Dependent Gating

Mitochondria are actively relocated to areas of high energy demand in the axon through movement along the axoplasm. The movement of mitochondria is regulated by the interaction of calcium-sensing trafficking machinery (such as Miro1/2 with TRAK1/2 adaptor proteins, kinesin-1 isoforms and the dynein–dynactin complex) with the localized activity in the axon, and by syntaphilin anchoring them to frequently active regions of the axon [79,80]. Recent studies utilizing high-resolution metabolic imaging techniques have shown that there are highly localized microdomains for NADH/NAD+, FAD/FADH2 and tightly controlled domains for ATP/ADP that regulate the rate of recovery of energy-intensive processes (such as Na^+^/K^+^ ATPase and SERCA) and limit the number of times the neuron can fire repetitively [81,82].

ER–mitochondria communication (for example, VAPB–PTPIP51) enables the regulation of the metabolic state of the mitochondria based upon the level of calcium entering the cell, and the mitochondrial fusion–fission program (for example, OPA1, MFN1/2, and DRP1) enables the adaptation of the shape and metabolic function of the mitochondria to changes in the activity of the neuron [83]. Furthermore, in addition to providing the necessary ATP for membrane functions, the mitochondrial lipid and cofactor pathways provide additional protection against oxidative stress; disruption of these pathways can result in a reduction in the safety margin for propagation and increased likelihood of failure under load [84,85].

On longer time scales, the biogenesis of mitochondria and the removal of damaged mitochondria (mitophagy) through potentially activity-regulated programs (such as PGC-1α) enable the refinement of the distribution and functional competency of mitochondria, which is consistent with the concept of enduring metabolic gradients that direct the temporal profile of conduction [86].

### 4.2. Myelin as a Slow Learning System and Axon–Glia Metabolic Synergy

Myelin provides a slow learning system that modulates the timing of conduction while supporting the metabolic needs of the axon. OPCs respond to activity through a variety of signals, including neurotransmitters, purineceptors and contact-mediated signals; activity patterns that are persistent can induce OPCs to differentiate into oligodendrocytes and myelinate previously non-myelinated tracts [87,88].

As they mature, oligodendrocytes adjust their parameters of conduction, including g-ratio, internode length, nodal spacing, paranodal seal formation and juxtaparanodal channel localization, through adhesion molecules (contactin, neurofascin-155, and Caspr) and thus constrain the location of channels and the dynamics of propagation [89]. At the same time, oligodendrocytes supply glucose/lactate/pyruvate/ketone bodies through monocarboxylate transporters (MCT1-MCT2) and nanostructures associated with the myelin sheath to stabilize axons during periods of prolonged activity [90].

Changes in the geometry of the sheath that occur over days to weeks as a consequence of experience-dependent remodeling enable millisecond-scale delay adjustments that support long-range synchronization and timing-dependent computation; mechanosensitive responses at the paranodes may also link structural and ionic contexts [91,92]. A summary of the axes of control of mitochondrial–myelin interactions is provided in Table 2.

### 4.3. Multiscale Integration of Structural, Electrical, and Metabolic States in Conduction Plasticity

Conduction plasticity arises from the interplay among mitochondrial microdomains, glial-supplied substrates, and myelin geometry. For myelinated segments, impaired oligodendrocyte lactate support or decreased expression of MCT1 can decrease the ability of neurons to recover ATP after repetitive spiking, prolong repolarization, and increase the likelihood of depolarization block [98]. In lightly myelinated or unmyelinated collateral branches, reliability is typically more sensitive to mitochondrial positioning and glial-supported glycolysis during rapidly alternating spiking regimes [103].

Nodes of Ranvier are locations where dense clusters of sodium channels converge and require the coordination of mitochondrial placement, glial-fueled lactate, and astrocytic potassium buffering (via Kir4.1 uptake) to maintain the ionic gradients that allow for successful spike regeneration [104,105].

The long-term maintenance of lipid homeostasis in myelin (sphingolipids, cerebroside, and cholesterol) can affect both the mechanical properties of membranes and the stability of paranodal junctions, and therefore influence conduction velocity and nodal excitability [106]. Overall, it seems plausible that propagation regulation will be multiphasic (rapid mitochondrial/ionic gating, intermediate glial metabolic support, and slow myelin remodeling) to allow reliable timing under varying loads [107,108].

The potential of the multiscale approach outlined in the report could also be explored through experimentation where each layer is characterized by different types of perturbation methods with measurable and observable phenotypic properties. As an example, normal functioning of axons and glial cells (i.e., axonal maintenance and supply of metabolites) for myelinated pathways can be selectively blocked through the use of monocarboxylate transporter blockers or inhibition of oligodendrocyte energy production [109]. Following the disruption of normal function of the myelinated pathways, stimulation of both the myelinated pathways and the lightly myelinated/unmyelinated collateral pathways can be performed with the same paradigm and measurements of conduction reserve (i.e., latency drift, temporal dispersion, refractory recovery, and depolarization block threshold) can be collected. Contributions of mitochondria to the function of lightly myelinated or unmyelinated collateral pathways can be determined by blocking either mitochondrial trafficking/anchoring or mitochondrial fission–fusion [110]. Once mitochondrial function has been determined, burst duration, recovery rates and fiber branch-selective dropout can be measured to determine how well these pathways will operate during states of metabolic stress. Regulation of astrocytes at the node may be determined through high-speed voltage imaging or multisite electrophysiology if perinodal K^+^ buffering and ion homeostasis are selectively disrupted while simultaneous measurement of periaxonal K^+^ accumulation and the probability of generating new action potentials is being collected. Lastly, the relationship between the lipidome of the myelin sheath and timing accuracy may be examined by selectively disrupting the lipidome and ultrastructurally measuring the g-ratio, nodal length, paranodal integrity, juxtaparanodal segregation and conduction delay in disease-relevant models of demyelination, ischemic injury, chemotherapy-induced neuropathy and traumatic axonal injury [111].

## 5. AI-Enabled Mapping, Modeling, and Mechanistic Discovery in Axonal Systems

The ability of artificial intelligence to contribute to axonal research has moved beyond simply providing descriptions of axonal characteristics, and instead now enables researchers to reconstruct axonal morphology at a scale encompassing all degrees of detail, synthesize data generated by multiple imaging modalities, and correlate the morphology of individual axons with their propagation phenotypes as a function of physical and environmental conditions [112,113]. Additionally, it is clear that the utility of artificial intelligence extends far beyond the automation of processes and, when combined with existing knowledge regarding the functioning of mechanisms and the definition of objectives, artificial intelligence can identify trade-offs in geometry, channel organization, energy availability, and developmental constraints on an axonal system, and generate testable hypotheses for the consequences of manipulating one or more specific components [114,115].

Pipelines employing deep learning algorithms have been created to employ data collected during connectome imaging to construct high-resolution, detailed representations of the long-distance axons contained in tera-scale datasets describing axonal morphology over a wide range of spatial scales (i.e., micrometers to nanometers), using feature extraction and long-range contextual attention to maintain the connectivity of axonal structures and quantify the ultrastructural properties of axonal morphology, including the variation in axonal caliber, the curvature of the axonal membrane, the density/orientation fields of the cytoskeleton, and the distributions of organelles [116,117].

The reconstruction of axonal morphology can also include data from multiple modalities using techniques such as EM with Raman spectroscopy, fluorescence redox mapping, and label-free structural imaging to establish quantitative correlations between the morphology of the reconstructed axon and a variety of other properties, including the degree of saturation of lipids in the membrane, the dielectric constant of myelin, the characteristics of the cristae of mitochondria, and the integrity of the endoplasmic reticulum [118]. Additionally, models trained on paired datasets from multiple modalities can be used to estimate the local molecular state of components of the axon (e.g., the composition of ion channels, the types of post-translational modifications present on tubulin, and the presence of assemblies associated with liquid–liquid phase separation) based on structural proxy signals, allowing researchers to identify relationships between structural phenotypes and functional constraints that are interpretable [119].

Similarly, the same types of frameworks can be used to investigate the “interactomes” of organelles, by reconstructing the contact regions between the ER and mitochondria, areas of capture by vesicles, and regions of alignment between microtubules and organelles, thus allowing researchers to transform static images into state maps that are more relevant to excitability and resilience than those that arise solely from geometric considerations [120]. Generative models treat axonal anatomy as a constrained design space. Diffusion-based generative models and topology-preserving GANs can be employed to generate realistic hierarchical branching topologies, fluctuation in diameter, distributions of cytoskeletal bundles, and arrangements of organelles, while simultaneously generating hypothetical morphologies that extend the range of possible hypotheses [121].

These models can be employed in conjunction with functional objectives (using either embedded simulators or metrics of conduction) to explore trade-offs that result from differences in design decisions (e.g., the trade-off between energetic efficiency and timing precision, and the trade-off between structural complexity and robustness under metabolic stress and stochastic channel noise), and propose candidate geometries that satisfy the specified set of constraints [122]. Additional extensions that generate developmentally plausible trajectories of the morphology of the axon under altered firing, mechanical forces, or metabolic limitations can be employed to discover attractor-like configurations and candidate conserved design rules that can be tested experimentally [123,124].

Physics-informed modeling increases the accuracy of mechanistic prediction. Physics-informed neural networks can be employed to simulate propagation behavior by incorporating cable dynamics, ionic diffusion, dielectric/mechanical parameters, and metabolic dependencies into a single model, which can be employed to simulate propagation behavior in regimes that are difficult to reproduce experimentally (e.g., channelopathies that induce minor effects, constrained energy supplies, and temperature gradients) [125]. Moreover, imaging-anchored “digital twins” that combine geometry with inferred molecular/organellar landscapes can be generated, and allow for controlled virtual alterations of the geometry of the paranode, the organization of microtubules, the spacing of mitochondria, and the features of myelin to predict the outcome of conduction and the paths of early failure [126,127]. Inverse modeling represents yet another means to determine the internal configurations (distributions of channels, arrangements of organelles, and geometries of myelin) necessary to generate observed conduction profiles, thereby converting phenotypes into constraints on hidden state variables [128,129].

Multiscale co-simulation creates reciprocal feedback between the slower axonal processes (e.g., mitochondrial redistribution, cytoskeletal remodeling, and myelin adaptation) and the faster network processes (e.g., spike statistics) to formalize relationships between spike statistics and propagation constraints [130].

Reinforcement learning builds on this relationship by treating axonal segments or branch points as adaptive agents that learn resource allocation or parameter tuning policies to meet timing, resilience, or efficiency objectives, resulting in emergent strategies that can be compared to biological signatures [131]. Multielement models can be employed to simulate populations of interacting axon bundles that share extracellular resources and ionic clearance to study emergent routing biases, fascicle-scale synchrony, and timing alignment under collective metabolic burden [132]. Together, the toolchains developed to enable researchers to study the structure and function of axons individually can be used to develop predictive pathology by identifying early signs of vulnerability (e.g., disorganization of the paranode/perinode, changes in the geometry of the node, displacement of mitochondria, and disruptions in the periodicity of the cytoskeleton) prior to degeneration becoming irreversible [133]. For example, in demyelinating diseases, slowed conduction velocity, temporal dispersion, and intermittent blockade can be used as pre-lesion indicators of risk [134]; in metabolic stress syndromes (e.g., mitochondrial dysfunction, chronic ischemia, diabetic and chemotherapy-induced neuropathies), models that incorporate redox reporters, distributions of organelles, and histories of activity can be used to predict instability markers (e.g., latency drift, reduction in waveform margin, increase in propensity for afterdepolarizations, and decrease in burst duration) [135,136].

Virtual perturbation (“in silico lesioning”) can be employed to discover the effects of candidate mechanisms (e.g., mitochondrial function, channel distribution, branch structure, or stability of the paranode) by selectively disrupting them, and examining compensatory capacity under defined loads—which is particularly useful for traumatic axonal injury with focal swelling and transport failure [137,138,139,140]. Because load can produce similar macroscopic blockage phenotypes, but may indicate whether the blockage represents load-driven or recovery-limited conduction failure, these simulations can help to isolate the mechanisms responsible for each type of failure [141].

Generative pathology models can then be used to simulate coupled progression trajectories (metabolic deficit, structural fragmentation, and electrodynamic drift) to generate early biomarkers that are mechanistically interpretable, and evaluate the effectiveness of interventions aimed at enhancing the resilience of axons by decreasing load, increasing energetic reserves, or stabilizing microdomains [142,143]. Unsupervised and self-supervised learning can be used to categorize voltage, ionic, metabolic, and mechanical measurements into latent conduction regimes (e.g., stable, drift-prone, pre-failure, recovery-limited, and redox-linked), estimate transition structure, and extract early warning features [144,145]. By identifying multivariate coupling relationships (e.g., geometry channel organization, ATP/redox-recovery kinetics, tension membrane stability), AI can generate hypotheses that describe both perturbations and expected propagation signatures (latency dispersion, failure probability, and branch selectivity) [146,147,148].

**Figure 2:**
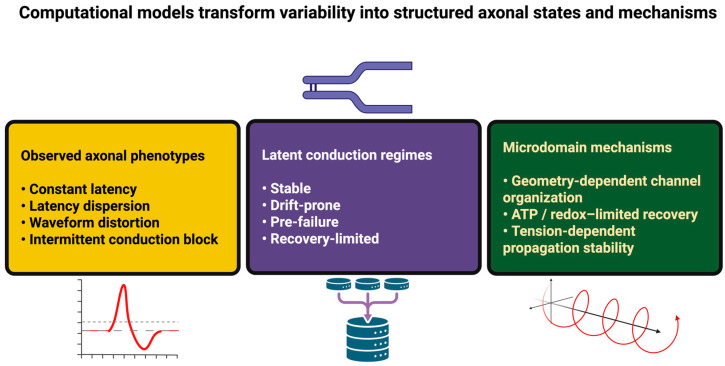
Computational identification of latent axonal states and microdomain mechanisms. Computational analysis links observable axonal propagation phenotypes to discrete latent conduction regimes and their underlying microdomain mechanisms. Apparent variability in latency, waveform, and conduction reliability is organized into structured states (stable, drift-prone, pre-failure, and recovery-limited), which are further resolved into distinct mechanistic causes, including geometry-dependent channel organization, energy-limited recovery, and tension-dependent propagation stability.

Inverse simulation can then be used to distinguish between mechanism classes that appear to be indistinguishable at low resolution (e.g., leak-dominated failure vs. energy-limited failure), and AI-assisted experimental design can be employed to select stimulation sites and measurement locations that will maximize the amount of information obtained regarding causal control variables (Figure 2) [149,150].

Together, the methodologies described above provide a complete framework for transforming large-scale mapping to mechanistic prediction and falsifiable intervention, and guiding the closed-loop strategies described in Section 6 [151].

## 6. Closed-Loop AI Interrogation, Adaptive Modulation, and Causal Reconstruction of Axonal Dynamics

Closed-Loop AI is transforming the way axons can be investigated experimentally. Instead of using only passive measurement techniques or predetermined stimulation paradigms, modern closed-loop AI systems measure axonal behavior in real-time; modify stimulus based on real-time axonal response; and develop internal models of axonal behavior based on real-time data obtained from every iteration of stimulation. Closed-loop AI effectively converts axons into dynamically controllable systems whose structural, metabolic, electrodynamic, and mechanical characteristics can be identified from controlled interactions [152]. Closed-loop AI achieves this goal through real-time control; real-time targeted modification of axonal microdomains; and through developing internal models of axonal behavior. Closed-loop AI identifies axonal regulation rules that would otherwise remain undetectable via static or open-loop measurement techniques. The application of closed-loop AI techniques enables the creation of causal maps of propagation dynamics; reveals hitherto unobserved internal states of axons; and provides insight into the processes whereby axons protect or compromise function in response to changes in external conditions [153].

### 6.1. Precision Perturbation and Real-Time Control of Axonal Microdomains

Beyond controlling the axonal signal through optical voltage imaging or by monitoring/controlling at the same time scale as the action potential itself, closed-loop systems enable the controller to utilize real-time information regarding the system’s state variables, such as voltage transients, Na^+^/Ca^2+^ oscillations, redox states of mitochondria, and nanometer-scale deformation of the plasma membrane, to modulate stimulation at the same time scales as the action potential propagation [154]. This represents a fundamentally new mechanistic understanding, from viewing the previously described phenomenon of latency jitter, transient blocking of action potentials, dropout of branches, and distorting waveforms as experimental error, to treating them as state transitions that may be altered by modifying stimulation within milliseconds to establish the causal boundary conditions for stable conduction [154].

One of the greatest advantages of the ability to close loops in order to experimentally explore axonal behavior is the fact that the reliability and routing characteristics of an axon are determined by ‘control points’; these control points are generally sparsely located throughout the length of the axon due to impedance discontinuities, compartmentalization of different types of channels, and differences in the loading of each branch. Stimulation paradigms that are spatially constrained (for example, nodal versus internodal targeting, collateral restricted stimulation, or stimulation confined to the branch point) will therefore enable researchers to determine where conduction is sensitive along an axon, maximize the safety factor against propagation failure, and determine the characteristics of the microdomain governing the outcome of the propagated signal [155]. Practically, these methodologies will enable researchers to differentiate between localized changes in excitability and localized determinants of conduction probability and delay—which is generally difficult to separate using traditional global stimulation techniques [156]. Another methodology researchers can employ to determine the mechanism of conduction in an axon includes localized ionic microdomain-level interventions [157]. The ionic environment surrounding the membrane and the periaxonal space can affect the propagation of the action potential; however, this occurs without producing significant alterations in the total conductivity of the membrane. Researchers have utilized several methods for employing ionic microdomain-level interventions to determine the mechanism of conduction in an axon, including the localized release of ions, through localized ionic perturbations, which can be employed to stimulate a particular location in the extracellular space or in the periaxonal space to determine the effect of localized increases/decreases in Na^+^, K^+^, or Ca^2+^ concentrations upon the development of waveforms, the latency of conduction, and the likelihood of an axon experiencing propagation failure as a direct consequence of prior activity [158]. Furthermore, closed-loop control of the ionic perturbations will afford researchers a means of rapidly obtaining the local transfer function—the mapping relationship between the ionic state of the microdomain and the phenotypic expression of the propagation—so that “failure” can be broken down into understandable mechanisms (load mismatch, limitation in clearance, etc.), rather than being treated as a singular event [159]. Additionally, researchers can apply precision perturbations to the conduction of an axon in ways that do not require the application of electrically controlled stimulation—since propagation is dependent upon the state of the substrate. For example, researchers can alter the mechanical properties of the substrate (pressure or optical force) locally to determine the areas of the axolemma and cytoskeleton that are sensitive to tension, whereas researchers can use focal photochemical modification of the lipid composition of the membrane to determine if changes in the viscosity, curvature, and microdomain organization of the membrane can affect excitability and stability [160].

These types of perturbation will enable researchers to separate the effects of kinetic properties of channels from those produced by the membrane–cytoskeletal environment that organizes channels and determines mechanosensitivity—thus affording researchers mechanistic insight beyond that provided by models that are based solely on voltage. By enabling researchers to close loops, researchers can increase their causal inference by developing individually optimized response maps for a particular axon, after repeatedly applying perturbations [161]. Closed-loop controllers can identify rapid regime shifts, identify microdomains that are crucial to the reliability of conduction, and determine how slight redistributions of ions, organelles, or membrane tension influence both the waveform and the path of the propagated signal. Conceptually, this will allow researchers to view an axon as a dynamically controlled system, in which the regulatory structure can be identified as a collection of local constraints and latent state variables describing the transitions between regimes of propagation that are either stable or failure-prone [162].

Typically, a closed-loop perturbation experiment is conducted in two stages. First, the conduction properties of a given system are measured using an open-loop method. Then, based upon the results obtained from the initial open-loop measurements, closed-loop perturbation protocols are created that will utilize closed-loop control systems to repeatedly attempt to modify the value of a candidate control point (i.e., node, paranode, internode, or branch point), to generate a relationship (or “transfer function”) of the state of the microdomain to its propagated phenotype [163].

For example, if you wanted to investigate the influence of alterations in the ionic environment within the microdomain on both the safety factor and regenerative capacity of action potential firing, you could combine an adaptive stimulation protocol with local uncaging of either K^+^ or Na^+^ ions as well as with localized disruption of the normal clearance of ions by the periaxonal space. The same methodology can be employed to assess the impact of targeted manipulations to the anchoring/transport of mitochondria and/or to the membrane tension on the minimum metabolic/mechanical margin(s) necessary for the maintenance of stable conduction, and thereby produce quantitative “resilience curves” from qualitative descriptions of success/failure during repeated stimulation bursts [164].

### 6.2. Adaptive Neuromodulation and Reallocation of Intracellular Resources

Closed-loop neuromodulation extends the scope of the experimental paradigm from brief, isolated perturbations by creating the possibility for sustained, adaptive interactions between closed-loop systems and axonal systems as they respond to environmental or imposed challenges. Using reinforcement learning, controllers can explore a large number of potential modulatory actions and adjust local ionic conditions; metabolic substrate availability; membrane mechanics; or receptor pathways to maintain conduction within a stable range. As the axon compensates through internal adjustments, the controller develops knowledge regarding which types of interventions will support stability and which will result in instability [165,166].

**Figure 3:**
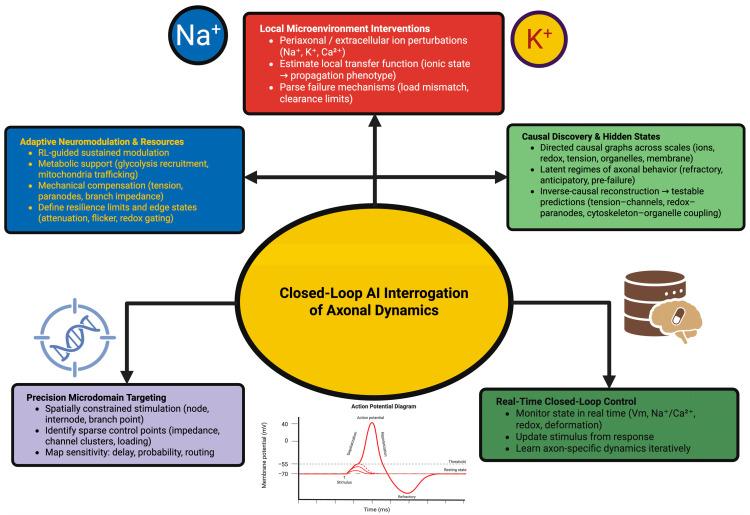
Closed-loop AI interrogation of axonal dynamics. Real-time sensing of axonal state variables (voltage, ionic flux, redox, and biomechanics) enables adaptive stimulation and precision perturbation of axonal microdomains. Iterative control reveals sparse conduction “control points”; maps transfer functions linking local microenvironment to propagation phenotype and define resilience boundaries under metabolic/mechanical stress. Causal discovery and latent-state modeling reconstruct directed mechanistic architecture and hidden propagation regimes, generating testable predictions of stable vs. failure-prone conduction.

As a result of this iterative exchange, closed-loop neuromodulation discloses the axon’s potential for self-regulation. During periods of metabolic stress, conduction may be preserved through localized recruitment of glycolytic assemblies or the movement of mitochondria toward high-demand regions. Under mechanical stress, axons exhibit temporary changes in cytoskeletal tension; paranodal geometry; or branch point impedance, all of which can be detected as slight deviations in the trajectory of conduction. Through high-resolution temporal monitoring, closed-loop neuromodulation illustrates the latent regulatory modalities through which axons maintain coherent signaling under dynamic internal conditions [167,168]. Gradually increasing the complexity or intensity of imposed challenges, adaptive systems can define the boundaries of axonal resilience. At the boundary of resilience, conduction becomes unstable, resulting in the appearance of spike attenuation due to localized microdomain dysfunction; variable gating due to redox driven phenomena; or flickering propagation that briefly switches between successful and failed conduction. The examination of these “edge states” provides insight into how axons distribute internal resources; coordinate organelle function; and temporarily rearrange the distribution of channels in an effort to preserve conduction fidelity. Examples of the multiple strategies available to axons to stabilize function and examples of the threshold values above which those strategies fail are provided [169]. Figure 3 summarizes the conceptual framework of closed-loop AI interrogation of axonal dynamics.

The logical validation process of these models’ applicability to closed-loop neuromodulation systems involves design elements explicitly included within the system to ensure translational relevance, i.e., randomized target sites for stimulation, sham perturbation stimuli, stimulation trains identical to those utilized in open-loop studies, and cross-validated controller policies [170]. A model of a disease can be developed by imposing controlled metabolic limitations on the axon, i.e., by limiting the amount of substrate available to the axon, by reducing the delivery of lactate by glia, by creating demyelinating-like conditions at the paranode (i.e., by making the paranodal region more loosely connected), by modeling traumatic injury to the axon, such as through focal swelling, which would generate impedance bottlenecks analogous to those generated by focal swellings, or disrupting transport [98]. The capability of determining whether the application of adaptive control provides an axonal “conduction reserve” versus simply shifting the site of action potential failure in the axon will directly relate the axonal microdomains responsible for the mechanism of action of a specific treatment to clinical observations, including, but not limited to, intermittent conduction block, temporal dispersion, and progressive decrease in the ability to sustain firing [171].

### 6.3. Causal Discovery and Reconstruction of Hidden Mechanistic Architecture

The most significant benefit of closed-loop AI is the ability to infer causality from experiments involving interactive interrogation. Through the integration of the effects of thousands of perturbations, causal discovery algorithms generate directed relationships between variables that describe ionic flow; cytoskeletal tension; redox activity; organelle placement; and membrane biomechanics. In many cases, causal discovery algorithms establish dependencies that would never appear from observation alone, including, for example, that certain mechanically sensitive regions of the axon regulate excitability in a load-dependent manner or that localized redox oscillations can condition mitochondrial responses that influence the reliability of subsequent spikes [172].

Latent-state models developed without prior supervision build upon these observations by clustering responses to perturbations into distinct internal states of axonal behavior. Some of the clusters correspond to familiar physiological states, while other clusters represent previously unrecognized states of axonal behavior such as transient structural refractory phases; metabolic anticipation periods before firing bursts; or intermediate excitability states that exist immediately before conduction failure. The latent-states provide a higher level description of the ways in which the axon transitions from one functional state to another in response to chronic stimulation or changes in the environment [173].

Inverse causal modeling provides additional insight into the underlying mechanism by reconstructing the minimum combination of parameters necessary to produce the observed patterns of propagation. Many of the reconstructed combinations of parameters suggest interactions that have not been experimentally confirmed, for example, tension-dependent modulation of specific families of channels; redox-dependent control of paranodal adhesion; or coupling mechanisms between the cytoskeleton and organelles that facilitate coordinated conduction under varying loads. These hypothesized interactions can be treated as experimentally testable predictions, and thus guide future research to discover molecular actors or structural elements that fulfill computed roles [174,175].

In summary, the integrated methodologies of closed-loop AI transform axonal experimentation into a cooperative endeavor in which the system being studied generates hypotheses about itself in real-time. Closed-loop AI discloses causal connections between electro-dynamic; metabolic; and structural levels of organization, illustrating how axons can maintain coherent signaling within a dynamic internal environment. The methodologies presented here form the basis for Section 7, where these ideas are expanded to include broader computational architectures and translational applications.

## 7. Computational Convergence, Neuromorphic Translation, and Cross-Domain Principles of Axonal Information Processing

An emerging framework exists for understanding how the functional characteristics of axons can be interpreted in terms of computational systems. Advances in both molecular biology and the use of imaging techniques have been combined with the application of machine learning algorithms and demonstrate that axons are not passive conduits but instead are adaptive computational substrates composed of interacting layers of structure, metabolism, and electrodynamics [112,176]. The computational frameworks developed using AI-driven reconstruction, generative modeling, and closed-loop interrogation provide the basis for reinterpreting the functional characteristics of axons in computational terms. The application of computational principles to the study of axons enables them to be viewed as having several properties of distributed information processing systems (i.e., local autonomy, multi-time scale state transitions, resource-dependent computation, and context-sensitive propagation), which also resonate with principles of artificial intelligence, neuromorphic engineering, and adaptive control theory [121]. Unlike relying on metaphorical interpretation, the convergence of axons with artificial intelligence and neuromorphic engineering occurs due to a convergence of fundamentally similar mathematical structures and regulatory motifs [144]. Therefore, Section 7 will describe the parallelism between axons and distributed systems, discuss the translational implications of the parallelism, and articulate a theoretical synthesis of axons as part of a larger class of adaptive computational systems.

### 7.1. Distributed Computation, State-Dependent Signaling, and the Emergence of Local Intelligence

Using high-resolution axonal datasets, AI models that exhibited computational motifs that are consistent with those found in distributed systems in machine learning were trained. At every point along the axon, a single axonal segment operates as a computationally informed unit that combines ionic gradients, mechanical forces, metabolic capacity, and organelle configurations. Transformation rules that depend on the internal state of the microdomain are contained in microdomains along the axon [177]. Therefore, the same inputs result in different outputs based on the condition of the microdomain. Architectures such as these are analogous to gated recurrent units or adaptive attention mechanisms, where slower variables modulate the rate of fast signal propagation [178].

Slow variables of the type described above exist in axons and include membrane curvature fields, redox oscillations, cytoskeletal tension, lipid-phase composition, organelle occupancy, and residual ionic microgradients. These slow variables are generated through dynamics that occur independently of the current spike train but significantly affect the propagation probability, timing drift, refractoriness, or conduction gain of the spike train. The structural and metabolic constraints that were discovered through AI form a secondary computational layer—a supervisory architectural layer that influences the mapping from input to output along the axon [179]. As a result of the interaction of fast spike dynamics and slow modulatory states, axons exhibit computational behaviors analogous to hierarchical controllers that utilize local mechanisms to correct errors, maintain conduction, and allocate energy resources under fluctuating conditions. Axons demonstrate context-sensitive routing, with selective amplification or suppression of propagation along certain collaterals based upon temporal history, metabolic state, and localized channel composition [180]. The degree to which axons exhibit local intelligence in the sense that they implement rule-based adaptive behavior without centralized oversight represents a significant departure from traditional nerve models and creates an avenue for developing theories of axonal function as a computational participant in network function as opposed to a passive intermediary between synapses [181].

### 7.2. Neuromorphic Translation, Computational Materials, and Axon-Inspired System Design

This section will explore the similarities between the biological functions and mechanisms of axon propagation and those found in neuromorphic engineering systems. Our ultimate goal is to develop a common understanding of the functions of biological systems and engineering systems alike through the interaction of their electrical, mechanical and thermal properties to achieve the desired functionality, and to identify the engineering-relevant design principles and concepts in the biological substrate that relate to the development of engineering systems based on our common understanding of how biological and engineered systems operate. There are many factors that restrict the ability of a signal to propagate along an axon and thus cannot be described using a simply defined electrical parameter, and therefore cannot be accurately modeled using a simple cable model. Local availability of energy, micro- and mesoscale geometry (branching, myelination, etc.), and electromechanical conditions all greatly impact the latency, safety margin, and failure probability of a spike transmission. There is therefore a natural connection between the biophysical processes that govern the transmission of a signal along an axon and the development of neuromorphic systems, since the biophysics of axons require that signal propagation is a state-dependent process, in which the overall performance of the system is governed by the combined effects of the biophysical properties of the substrate and the spatiotemporal pattern of electrical activity versus the conductivity of the surrounding medium [182].

Since spike transmission depends on the continuous replenishment of ionic gradients and the maintenance of membrane potentials between spikes, the local ability to produce ATP will determine the range of firing rates possible by determining the rate of recovery and the effective safety margin for conduction. The spatial localization of ATP production and the redox state (determined by the position of mitochondria and the existence of local metabolic assemblies) will determine the operation of pumps and therefore the duration of the refractory period, excitability threshold, and susceptibility to activity-dependent reductions in the fidelity of conduction [183]. Therefore, this is an example of metabolic gating in the biological sense: propagation will be reliable as long as there are sufficient local energetic resources to sustain it; when such resources are no longer available, propagation will either occur probabilistically or fail completely. Thus, this provides a basis for translating the neuromorphic representations in which the energy state does not constrain propagation to the internal variable that dynamically influences the accuracy of transmission, burst duration and routing, and therefore the system will demonstrate a controlled degradation or an adaptive redistribution of resources rather than complete collapse [184].

As mentioned earlier, geometry will play a large role in computing within axonal signaling. Variations in impedance at branch points, diameter and in the division of the axon into nodal and internodal sections will vary the capacitive loading and current partitioning as a function of location and therefore influence the progression of action potential waveforms and the reliability of conduction in collateral branches [41,71,185]. Additionally, in myelinated systems, the length of internodes and the dielectric properties of the myelin sheath will provide an additional level of temporal control over propagation delays and synchronization independent of synaptic plasticity. Thus, we suggest applying an engineering principle of thumb wherein computation can be performed through structural parameterization—through the distribution of impedance gradients and tunable delay lines—so that information processing can be achieved through the modification of geometric and material properties as well as through the modification of conductance-like weights [186].

Additionally, axons present a substrate-level view of computation in which mechanical state variables interact with electrical dynamics. While voltage is commonly considered the primary signaling variable, electromechanical coupling and a mechanically sensitive microstructure can modify the effective excitability and stability of propagation by modifying membrane tension, organizing channels based on curvature, and modifying local dielectric/membrane properties. Therefore, the key point of translation is not to assign human-like intelligence to axons as “smart materials”, but to recognize that axon function is contingent upon the coupled physical degrees of freedom [187]. Thus, this introduces the concept of computational materials, in which physical state (e.g., strain, morphology, and phase state) can modify or reorganize signal propagation, and therefore create substrates that can be reorganized so that certain aspects of the computation are encoded in the evolution of the physical configuration of the substrate itself [188,189].

Biologically, axons exist at multiple time scales, and the slow variables can drive an axonal segment into a variety of different functional regimes. Prior to the onset of activation, the metabolic state, microstructural organization, and previous load history can cause a transition from a high-fidelity transmission regime to a regime characterized by drift-prone, delay variable, or intermittently failing modes of operation in propagation, regardless of whether the driving spike train is identical. Therefore, if we were to interpret this formally, this indicates that the system demonstrates a number of internal states that are latent and modulate the input/output mapping between incoming spikes and downstream propagation results [190]. Therefore, in designing neuromorphic systems, this implies that the use of explicit internal state variables (metastable modes or state machines) that define the operating regimes (reliability prioritized vs. energy prioritized) in which the system operates and allow for context-dependent routing and stability control in the presence of noise should be used [191].

Consistent with biology, a synthesis of the above is that axonal conduction is due to an equilibrium among the energetic supply, structural constraints and electrical load. When the equilibrium is favorable, then propagation occurs reliably with tight latency control; when it is unfavorable, then conduction becomes probabilistic or fails [192]. Conceptually translating this implies that robustness should be viewed as a control problem: maintain functional signaling through regulation of the coupled energy–structure–load equilibrium, rather than optimizing an electrical property independently [193].

In summary, models of axonal electromechanics have shown that redundancy in multidomain systems can contribute to resiliency: disruptions in one layer (electrical, mechanical or energetic) can compensate for disruption-induced responses in other layers. Therefore, engineered systems can exhibit increased stability if computation is distributed across interacting physical layers, rather than being confined to a single substrate, thereby providing a method for gradual adaptation to disruptions that would otherwise destabilize homogeneous architectures [194].

### 7.3. Cross-Domain Theoretical Parallels, Integrative Mechanisms, and the Foundations of Axonal Intelligence

The intersection of axonal biology with artificial intelligence and adaptive systems theory creates opportunities for developing integrative models of axons as multilayer dynamical systems that produce computation from the interactions among physical, chemical, and informational processes. Analogous properties of hierarchical time scales, local feedback, energy-dependent computation, and internally produced memory states exist in multiple systems that maintain stable operation under fluctuating constraints—including biological neurons, neuromorphic hardware, adaptive materials. Moreover, axons exhibit these properties across all species and brain regions [195]. Thus, theoretically, axons represent a class of systems where computation arises from constrained optimization. Constraints on electrical signaling imposed by biological considerations include trade-offs that are determined by metabolic availability, structural integrity, organelle capacity, and spatial organization. Optimization problems of similar types are also encountered in reinforcement learning, where policies emerge from optimizing behavior under constraint, and optimal control theory, where stability must be maintained despite uncertainty. The axonal regulation of conduction velocity, excitability, or routing represents the solution to a high-dimensional optimization problem that was formed by evolutionary and developmental pressures and solved by axons [196,197].

Inferences made possible by AI methods indicate that axons operate within hidden regulatory manifolds, where slow variables define a low-dimensional structure influencing rapid spike propagation. Manifolds of this type are analogous to the latent spaces of deep generative models, where complex data distributions collapse onto lower-dimensional internal representations. Regulatory manifolds of axons may serve as internal regulatory landscapes that stabilize electrical function in the presence of fluctuations in geometry, activity, or metabolic availability [198]. Together, the similarities between axons and other computational systems establish the basis for a unified theory of axons as adaptive computational media that combine electrodynamics, structural mechanics, metabolic flux, and localized biochemical networks to generate coherent signaling behavior. This viewpoint does not consider axons as engineering analogs; rather, it recognizes that biological systems and artificial systems converge on similar solutions to equivalent computational problems [199].

## 8. Conclusions

New breakthroughs in molecular neuroscience; structural imaging; electrophysiology; and artificial intelligence have shown the axon to be much more dynamic and integrative than ever imagined before. The current body of evidence reviewed here shows that axons modulate electrical activity through the coordination of metabolic microdomains, structural architectures, organelle dynamics and adaptive electro-mechanical processes. These findings suggest that axons do not simply transmit information but shape it—moment-by-moment—based on internal states and limitations of the axon itself. As researchers continue to develop new analytical techniques and methods to perturb (manipulate) systems to explore new layers of complexity, the axon has emerged not only as a supporting structure for synaptic transmission but also as an active, computational participant in neural circuits.

In organizing this review, we attempted to integrate multiple, rapidly developing areas of research into a single framework that appreciates the diversity between different disciplines and emphasizes their relationships. Our goal was to present a framework for integrating these areas of study that would be useful for guiding future research efforts rather than attempting to make conclusive statements about phenomena that remain poorly understood. Many of the mechanisms described here (such as metabolic gating to closed-loop adaptive behavior) require further experimental validation, and many of the theoretical models used to describe these mechanisms will likely undergo significant changes as more experimental data become available. We believe that presenting these concepts together will allow researchers to generate hypotheses that integrate molecular mechanisms with physiological function and computational implications. The role of artificial intelligence will be pivotal in this direction. AI-based reconstructions, multimodal integrations, generative morphological modeling, physics-informed simulations, and closed-loop perturbations have provided investigators with pathways to explore that were previously unavailable. These methodologies have allowed investigators to discover characteristics of axonal organization that cannot be observed using conventional methods alone and have provided investigators with a mechanism for identifying unknown relationships, proposing mechanistic explanations, and designing experiments with greater precision. Our purpose was to demonstrate how researchers can use such tools—when combined with an understanding of biology—to accelerate the discovery of principles that govern axonal behavior at all levels.

Although the scope of the growing literature is broad, we recognize that the work presented here represents only a limited survey of the increasingly diverse field of research. Important unanswered questions include: How do axonal processes interact with dendritic and synaptic processing? How do metabolic and structural adaptations affect the timing of network level processing? And how do diseases disrupt the fine balance required for reliable propagation? These outstanding questions emphasize the need for continued investigations that are supported by both experimental innovations and computational theories. Our intent with this conclusion is not to represent the culmination of knowledge but rather to extend an invitation for further inquiry. If the axon is a distributed, adaptive and computationally competent system, then there are many fundamental aspects of its functioning that have yet to be discovered. We hope that the framework we have developed in this review will serve as a starting point for future research, encouraging work that explores the mechanisms of axonal function and extends our understanding of how neurons process information, adapt to changing conditions and maintain reliable signaling over time.

As the field develops, we anticipate that future research will build on, challenge, or refine the perspectives we have discussed here. In addition, the axon continues to show complexities that inspire curiosity rather than completion, and it is our expectation that the ideas we have expressed in this paper will contribute, however modestly, to this ongoing scientific discussion.

## Figures and Tables

**Table 1:** Each regulatory axis captures a discrete mechanism through which axons construct self-sufficient microdomains. Localized translation systems provide differential decoding and proteomic heterogeneity; cytoskeletal patterning encodes directional transport and long-term structural memory; organelle–cytoskeleton coupling links redox, lipid exchange, and Ca^2+^ transfer to spatial organization; and condensate-based architectures generate ultralocal biochemical logic.

Axonal Regulatory Axis	High-Resolution Microprocesses	Emergent Functional Capacities	Core Molecular Systems	References
Localized Translation Ecosystems	Axon-specific ribosomes with selective rRNA/PTM signatures; spatially heterogeneous tRNA charging/modification; activity-tuned RNA granule phase states; microtubule-guided mRNA trafficking	Rapid, compartmentalized synthesis of excitability/metabolic regulators; autonomous protein supply during stress or injury	RPL/RPS variants; PUS enzymes; TRMT61A/10C; FMRP; hnRNPs; G3BP1	[52]
Translation-Gating Microarchitecture	Submicron translation “on/off” zones via mTOR/eEF2K/AMPK gradients; Ca^2+^ microdomains dictating ribosome recruitment; metabolic gating of elongation	Proteomic mosaics along single axons; energy-matched synthesis; localized response to firing patterns	mTOR nanoclusters; eEF2K; AMPK; Orai/IP_3_R; L-type Ca^2+^ channels	[53,54]
Microtubule Identity Encoding	Isotype mosaics creating mechanical anisotropy; PTM striping (acetylation, detyrosination, Δ2, polyglutamylation) as directional motor tracks; activity-driven PTM rewriting	Precision cargo routing; structural memory of electrical/metabolic history; regional cytoskeletal tuning	TUBB/TUBA isotypes; TTLL7; HDAC6; vasohibin–SVBP; kinesin-1/3; dynein	[55]
Spectrin–Actin Periodicity	Adjustable ring spacing via spectrin turnover; myosin-II tension gradients; Arp2/3 actin patches as anchor/branch sites	Elasticity control; localized vesicle dynamics; tension-adapted stability of conduction	αII/βII spectrin; adducins; myosin-II; Arp2/3; ERM linkers	[56]
Neurofilament Conductive Architecture	Phospho-tuned neurofilament spacing; Ca^2+^/kinase-regulated filament transit; slow accumulation of NF-H/M modifications	Fine conduction velocity tuning; long-term encoding of load history; region-specific caliber modulation	NF-H/M/L; Cdk5; CaMKs; phosphatases	[57]
Organelle–Cytoskeleton Interface Hubs	ER curvature microdomains; dynamic ER–mitochondria tethering; cytoskeleton-based organelle positioning	Local ATP/redox control; spatial regulation of vesicle fusion and branching; metabolic–structural integration	Reticulons; atlastins; ORP family; MAM components; Rab/KIF adaptors	[58]
Phase-Separated Signaling Condensates	Rapid kinase-scaffold condensates; liquid–gel switching of RNA granules; nanoscale lipid-modifying assemblies; metabolic enzyme clusters shaping ATP microgradients	Ultralocal biochemical computation; precise signal amplification/damping; energy–electrical alignment	CAMKIIδ; ERK/PKC; FUS/TDP-43; G3BP1; PI4KA/PLCβ3; glycolytic clusters	[59]
Mechanochemical Memory Encoding	Stable PTM landscapes from firing/mechanical load; tension-dependent condensate remodeling; cytoskeleton–organelle alignment as persistent “tracks”	Predictive structural adaptation; autonomous tuning of excitability/metabolism; long-lived compartment identity	HDAC6/PTM enzymes; spectrin variants; myosin-II; ER–mitochondrial tethers	[60]

**Table 2:** Each regulatory layer highlights a distinct mechanism through which metabolic resources and myelin structures influence signal propagation.

Regulatory Layer	Core Microphenomena (Ultra-Dense)	Propagation-Level Effects	Key Molecular/Cellular Systems	References
Mitochondrial Spatial Logic	Ca^2+^-gated arrest/transport cycles; redox-structured NADH/FAD microgradients; ATP/ADP nanodomains; fusion–fission–cristae reconfiguration tied to firing	Segment-specific energetic gating; spike frequency-dependent safety factor modulation; probabilistic vs. deterministic conduction transitions	Miro1/2; TRAK1/2; KIF5 isoforms; dynein–dynactin; syntaphilin; OPA1/MFN1/2/DRP1; VAPB–PTPIP51	[93]
Energetic Microdomain Architecture	Submicron oxidative phosphorylation loci; glycolytic condensates forming ATP “hotspots”; oxygen- and substrate-sensitive redox oscillations influencing ion-channel gating	Localized modulation of Kv/T-type Ca^2+^ channels; tuned Na^+^/K^+^-ATPase and SERCA performance; firing pattern-specific conduction reliability	Glycolytic clusters; LDH–GAPDH complexes; SERCA; Na^+^/K^+^-ATPase; redox-sensitive channels	[94]
ER–Mitochondria Coupled Metabolic Coding	Tether-regulated Ca^2+^ pulses; lipid-exchange microcircuits; coordinated mitochondrial boosting under repetitive activity	Activity-matched ATP output; enhanced recovery during high-frequency bursts; increased susceptibility to depolarization under overload	VAPB–PTPIP51; ORP lipid carriers; IP_3_R–RyR clusters	[95]
Biogenesis–Mitophagy Metabolic Memory	Activity-encoded mitochondrial renewal; long-range patterning of high- vs. low-demand zones; targeted excavation of energetically “silent” mitochondria	Progressive shaping of conduction profiles; metabolic imprinting of frequently used pathways; sustained endurance of projection axons	PGC-1α pathways; axonal autophagy machinery; Parkin–PINK1	[96]
Myelin Learning Dynamics	OPC decoding of patterned spikes; Ca^2+^ signaling microdomains; activity-dependent oligodendrogenesis; adaptive control of internode geometry (g-ratio, length, and paranodal tightness)	Precise tuning of conduction latency; long-term synchronization of distributed circuits; adjustment of propagation timing for sensorimotor and cognitive precision	AMPA/NMDA receptors (OPCs); P2X/P2Y receptors; ephrins; neurofascin-155; Caspr–contactin complexes	[97]
Glia–Axon Metabolic Exchange	Lactate/pyruvate/ketone shuttling; paranodal ER- and CNP-positive channels; connexin-based metabolite corridors	Energetic stabilization during sustained high firing; mitigation of Na^+^ accumulation and conduction failure; resilience in long-range fibers	Oligodendrocyte MCT1–axonal MCT2; connexins; CNP+ channels; astrocytic metabolic regulators	[98]
Myelin Lipid Dynamics & Dielectric Tuning	SREBP-driven lipid synthesis; activity-regulated sphingolipid/cholesterol remodeling; paranodal loop stabilization	Modulation of membrane stiffness and dielectric constants; channel localization fidelity; nodal excitability precision	SREBPs; cerebroside/sphingolipid enzymes; cholesterol regulators	[99,100]
Perinodal Ion–Metabolic Coordination	Astrocyte-mediated K^+^ clearance; glial tuning of periaxonal ionic microenvironments; alignment of mitochondria with node demand	Spike regeneration fidelity; protection from depolarization block; precise shaping of high-frequency conduction	Perinodal astrocytes; Kir4.1; Na^+^-channel clusters; mitochondrial node alignment complexes	[101]
Multiscale Conduction Calibration	Integration of fast metabolic gating + intermediate glia–axon exchange + slow myelin remodeling; cross-scale feedback loops	Long-term optimization of conduction velocity, timing precision, and endurance; circuit-level synchrony adjustment; dynamic balance between energy cost and information fidelity	Mitochondrial networks; oligodendrocytes/OPCs; astrocytic endfeet; myelin structural machinery	[102]

## Data Availability

The data presented in this study are available on request from the corresponding author.
